# Is Life Force Matter?

**Published:** 1887-03

**Authors:** J. Hardman

**Affiliations:** Muscatine, Iowa


### Is Life Force Matter?

BY J. HARDMAN, D.D.S., MUSCATINE, IOWA.

Read before the Muscatine Academy of Science, Monday Eve., January 10, 1887.

Biology is usually defined by lexicographers as the science of life, an investigation of what is termed life force and organic structure in nature.

All matter is pronounced as being animate or inanimate, organic or inorganic.

All matter then possessing structural form and developing by what is called growth, whether vegetable or animal, is classed as organic matter and is possessed by this mysterious force we call life, vitality, mentality, energy, etc.

Matter not thus organized, but held together by what are termed mere physical forces, as gravity, cohesive, capillary, or chemical attraction, etc., is inorganic and includes all material in nature not found in the vegetable or animal kingdoms.

The question that at first confronts us is, what is life? What is vitality, or this force, the evidence we appreciate by our natural physical senses, in ourselves and in all animated beings, whether flora or fauna? We observe its phenomena and variety and its influence, but of its real character, constitution, origin, or destiny we know but little. And why we know less of its true character than we do of gravity, of chemical law, etc., may it not be because we investigate it less?

Your reader appreciates his inability to do justice to this subject commensurate with the respectful attention you are willing to bestow, and feels more like shifting the duty upon other members of your committee. At most, we can but open the question at this time and be more ready to hear than speak.

At the threshold of this question we are conscious of the needed familiarity of all the sciences as aids, especially histology, embryology, chemistry, physics, physiology, and psychology.

In dealing with this question we must regard every factor as belonging to matter in some form or other. That to attempt to discuss an element of nature, and at the same time regarding it as an immaterial product, is in itself absurd, and our efforts certainly will prove futile.

Then, interrogatively put, *is life matter?* We think the biologist is forced to answer in the affirmative. The forces in nature we call gravity, cohesion, chemical attraction or affinity, electricity, etc., are mysteries as great relatively as is life force; and we are sometimes almost constrained to pronounce that there is but little mysterious difference between organic and what we call inorganic matter. In the generally accepted theory termed atomic we are confounded in mystery to know why a certain number of atoms of one simple element will unite with a certain number of atoms of another element or other elements to form a molecule of a new compound or matter; and further, that this mode and aggregation of molecules forms all bodies everywhere in existance, and that these molecules are in constant or perpetual motion.

There is nothing more strange about a life cell, either in vegetable or in animal growth, than is hidden in the apparent inanimate crystal. A mere spark of electricity passed through a solution will arouse our astonishment at the apparent instant creation of beautiful crystals of mathematical proportions, and after their own kind in structure, etc. We ask is there not life there? Something as mysterious, when an electrical impulse is made upon one end of a wire cable and felt at the other a thousand miles away?

The term materialism means more than we ever anticipated. The correlation of forces in nature must be in harmony with transmutation of matter, or in other words, varied conditions of matter change and modify its properties and influences upon matter, and thus establish what to us, with such limited abilities, seems a non-substance, or as we call it, an immaterial spirit.

Permit us then to trace a single familiar substance through a first, second, and third condition, and note the changes and some of the results.

Water as a solid (or ice) is quite comprehensible to our external or physical senses. We can feel it, raise it, taste it, separate it, etc., but we are not able to comprehend as to how the molecules adhere so firmly to each other; while, however, we add a few degrees of temperature it passes into a second condition, becoming a liquid. And now these same molecules move so readily upon each other, and what a great change in its characteristics and in its influences upon other substances! While a solid its virtues were comparatively locked, but as a liquid it becomes an indispensable agent in the process of nature, and without it animal and vegetable being would be an impossibility. We, however, add still a few more degrees of temperature, and it passes into a third condition or gaseous state, and now it differs almost wholly from what it was in either of the two former conditions, and its influence upon matter has greatly changed. It now loses weight, is invisible to the natural sight, is greatly enlarged in its dimensions, but the molecules are the same, composed of the same number of atoms of hydrogen and of oxygen.

And while we contemplate these great modifications, as this same substance in passing through these three conditions, viz., solid, liquid, and gaseous, what may be the result, or its conditions and capabilities, after it enters into the fourth condition, or as it is termed, ether?

Matter in this condition is thought to pervade all nature, not only what we call empty space, but exists in and with all matter in the lower three conditions. It has now become so attenuated that it is lost, or out of the reach of our natural physical senses to investigate! We find we can follow it but little further, save by inductive or hypothetic reasoning.

Then if capable of passing from a first condition to a second condition, and from a second condition to a third, why may it not surely pass from a third into a fourth condition, and from a fourth into a fifth, and so on, *ad infinitum?*

While we contemplate the obvious changes, influences, and effects of matter as presented and manifest in the three lower or more crude conditions, can mortal man divine what takes place where it is found in a higher and a still higher sphere or condition? May it not then appear or exist as *force*, and even as *life?* It certainly seems more probable than doubtful.

If the sixty-odd simple elements of matter that have been identified are susceptible of combining in so many varied proportions and ways to form the innumerable bodies, objects, and substances in the universe as the chemist recognizes them in the crude or lower conditions, what may be the incomprehensible possibility of results that may be going on in this higher or more attenuated state of matter?

To philosophise thus is not mere idle speculation, but it is opening up the entrance to a large and comparatively unexplored field for thoughtful investigation.

Prof. J. S. Cassidy, of Covington, Ky., in a discourse entitled "Matter–Spirit," says:

"It is with gases that Dr. Crookes has succeeded in demonstrating, by a series of magnificent experiments, the undoubted existence of a 'fourth condition of matter,' and it is not taking an iota from the grandeur of his researches to say, for he himself acknowledges, that Faraday, more than sixty years before, suggested on purely theoretical grounds, the probability of 'radiant matter as far removed from vaporization as that is above fluidity.'"

He quotes Dr. Crookes as follows:

"We have in these researches actually touched the borderland where matter and force seem to merge into one another; the shadowy realm between the known and unknown, which for me has always had peculiar temptations. I venture to think that the greatest scientific problems of the future will find their solution in this border-land, and even beyond; here, it seems to me, lie ultimate realities, subtle, far-reaching, wonderful."

The Professor adds in his own words:

"Is it not reasonable to infer that inasmuch as there is a fourth or ultra, condition now recognized by all—though unwillingly by many—as actual matter, there may also be a point were matter merges into the 'borderland' of the immortal? The fact that there is a form of force not yet discernable, but acknowledged by scientists, as being ever present, is suggestive of the future possibilities which science may accomplish in the unknown domain of spirit. If mind itself be merely a mode of motion, may not instruments of precision assist the rapidly developing science of biology, or the yet gloomy field of psychology in illuminating beyond—aye, beyond!—the point where what we conceive as matter ceases to exist?"

---

LIFE says that at last we know why "uneasy lies the head that wears a crown." A newly arrived chiropodist from the old country announces himself as late corn doctor to the court of Germany, and tells us he has removed corns from several of the crowned heads of Europe.

